# Postoperative Echocardiographic Reduction of Right Ventricular Function: Is Pericardial Opening Modality the Main Culprit?

**DOI:** 10.1155/2017/4808757

**Published:** 2017-05-14

**Authors:** Marco Zanobini, Matteo Saccocci, Gloria Tamborini, Fabrizio Veglia, Alessandro Di Minno, Paolo Poggio, Mauro Pepi, Francesco Alamanni, Claudia Loardi

**Affiliations:** ^1^Department of Cardiology and Cardiac Surgery, Centro Cardiologico Monzino, IRCCS, University of Milan, Milan, Italy; ^2^Unit of Metabolomics and Cellular Biochemistry of Atherothrombosis, Centro Cardiologico Monzino, IRCCS, Milan, Italy

## Abstract

Echocardiographic reduction of RV function, measured using TAPSE, is a well described phenomenon after cardiac surgery. The aim of the present study was to investigate the relation between the modality of pericardial opening (lateral versus anterior) and the postoperative right ventricular systolic function by comparing echocardiographic parameters in patients undergoing minimally invasive or traditional mitral valve repair. 34 patients with severe mitral regurgitation due to mitral valve prolapse underwent traditional (sternotomy) operation (Group A) or minimally invasive surgery with right anterolateral thoracotomy (Group B). A postoperative TAPSE fall was found in both groups. Group A experienced a significant postoperative TAPSE fall versus Group B with *p* < 0.0001.

## 1. Introduction

The importance of the right ventricle as a determinant of exercise capacity and its significant prognostic value in the evaluation of surgical outcomes have been largely proven [1, 2]. The decrease of two-dimensional indexes of right ventricle systolic performance after cardiac surgery is a well-known phenomenon [3] and it has been shown to already occur during [4] and immediately after [5] the intervention. Its recovery to basal values is often incomplete and an echocardiographic dysfunction can persist one year after the operation [6].

The pathogenesis of such event is disputed and several hypotheses have been suggested, including type of cardioplegia [7], myocardial hypothermia [8], cardiopulmonary bypass [9], pericardial adhesions [10], and the simple opening of the pericardium [11].

Two-dimensional echocardiography has limitations in evaluating right ventricle performance. Three-dimensional echocardiographic images have been recently introduced in order to better assess the right ventricle contraction. Most of the studies failed to show significant postoperative right ventricle three-dimensional functional changes despite the two-dimensional indexes' concomitant decrease [3].

The aim of the present study was to investigate the importance of the modality of pericardial opening (lateral versus anterior) on postoperative right ventricular systolic function by comparing two- and three-dimensional echocardiographic parameters in patients undergoing minimally invasive or traditional (full sternotomy) mitral valve repair.

## 2. Methods

### 2.1. Population and Study Protocol

Written informed consent to participate in this observational study, which was approved by Centro Cardiologico Monzino Institutional Review Board, was obtained from all patients. The study protocol conforms to the ethical guidelines of the Declaration of Helsinki as reflected in a priori approval by the institution's human research committee.

The population consisted of 34 consecutive patients (mean age: 52 ± 12 years; 27 males/7 females) with severe mitral regurgitation due to degenerative mitral valve prolapse. All patients were scheduled for isolated surgical mitral repair with traditional sternotomy (Group A, 17 pts) or minimally invasive approach (limited right anterolateral thoracotomy, Group B, 17 pts). Clinical and echocardiographic baseline patients' characteristics are shown in Table 1.

Exclusion criteria were urgent intervention, atrial fibrillation, inadequate echocardiographic acoustic apical window, tricuspid regurgitation superior to 1 degree (scale 1 to 4), associated procedures, mitral valve replacement, major pulmonary diseases justifying a right ventricular dysfunction, pulmonary hypertension, previous cardiac surgery, and preoperative left ventricular ejection fraction < 40%. Two-dimensional transthoracic echocardiography was performed before surgery and 6 months after surgery. The Local Ethics Committee approved the study and informed consent was obtained from each enrolled patient.

### 2.2. Surgical Procedures

Traditional mitral valve repair was performed via midline complete sternotomy; the pericardium was opened anteriorly with a reversed T incision. Standard cardiopulmonary bypass was performed with ascending aortic cannulation and two venous cannulas. Buckberg protocol myocardial protection was adopted.

Minimally invasive mitral valve repair consisted in a limited (5 cm) right anterolateral thoracotomy. Three accessory incisions (1 cm) were made for aortic clamp, left atrial retractor, and camera insertion.

Pericardial incision was lateral just above the phrenic nerve. Femoral artery and vein were cannulated for cardiopulmonary bypass. A single anterograde dose of Custodiol cardioplegia was administered.

All operations were conducted in moderate hypothermia (32°C); in all patients, a left atriotomy was made to access the mitral valve; different surgical repairing techniques were employed depending on the primitive mitral lesion but a prosthetic ring was implanted in all patients (Table 2). After completion of the surgical procedure, the pericardium was closed with a continuous suture line in both groups.

### 2.3. Echocardiographic Measurements

All echocardiographic examinations were performed with a Philips ultrasound system (iE33, Andover, MA, USA). Complete standard M-mode and two-dimensional echocardiographic examinations were performed according to the clinical laboratory practice using an S5-1 sector array probe. Left ventricular end-diastolic and end-systolic volumes, as well as biplane ejection fraction, were measured from apical four- and two-chamber views by the area-length method. Systolic pulmonary arterial pressure was noninvasively obtained using Doppler echo method from the systolic right ventricle-right atrial gradient, calculated from the systolic trans-tricuspid regurgitant flow peak velocity by the modified Bernoulli equation. Right atrial pressure was derived by means of the inferior vena cava collapsibility index measured from the subcostal view [12]. To evaluate tricuspid annular plane systolic excursion (TAPSE), defined as the difference in the displacement of the right ventricle base from end-diastole to end-systole, from the apical four-chamber view, the M-mode cursor was positioned at the junction of the tricuspid valvular plane with the right ventricle free [13, 14].

To evaluate right ventricular function, we followed the method of Tamborini et al. [3] Real-time three-dimensional transthoracic echocardiography was performed immediately following the two-dimensional examination, by utilizing an X3-1 matrix array probe. The three-dimensional datasets were acquired in the “full-volume” mode from the apical view, adapted to improve the visualization of the right ventricular chamber. Two datasets per patient were obtained and stored. Offline postprocessing and three-dimensional reconstruction were performed with a commercially available dedicated system (Echo View, Tom Tec Imaging Inc., Munich, Germany) equipped with four-dimensional right ventricle analysis software. This software is based on the manual tracing of the right ventricle endocardial contours in the end-diastolic and end-systolic frames performed in the sagittal, four-chamber, and coronal views, obtained by slicing the acquired 3D dataset [15]. Once manual initialization was completed, a semiautomated endocardial border detection algorithm was applied throughout the heart cycle. After manual correction, right ventricular end-diastolic and end-systolic volumes were automatically calculated. Then, right ventricular stroke volume and ejection fraction were measured as the difference and the percentage of change of the volumes, respectively.

### 2.4. Statistical Analysis

Data were collected and managed in Microsoft Excel 2003 and analyzed with SPSS 12.0 software (SPSS Inc., Chicago, IL). Doppler and two-dimensional and three-dimensional echocardiographic parameters of right ventricular dimensions and performance were evaluated before and 6 months after surgery.

Continuous variables were presented as mean ± standard deviation and compared with an unpaired* t*-test, while categorical data were expressed as percentages or numbers and compared with *λ*^2^ test.

A between-groups comparison examining the impact of the surgical technique on right ventricular function over time was made with an analysis of variance and covariance adjusted for patients' age, sex, body surface area, and right ventricular features basal values.

A *p* value < 0.05 was considered statistically significant.

## 3. Results

**Figure 1:**
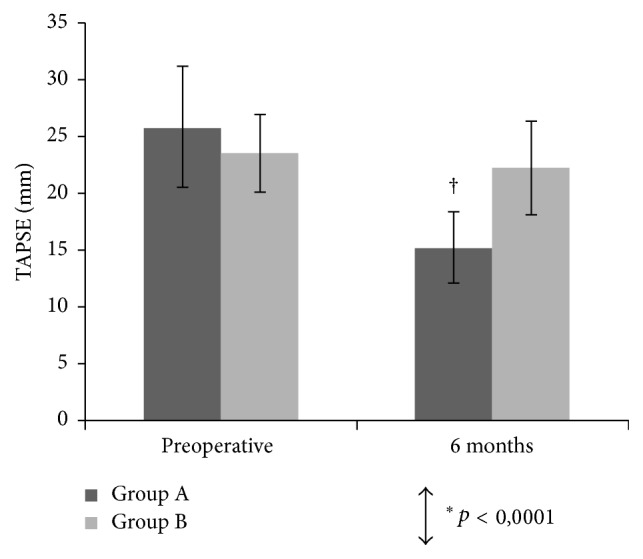
Mean tricuspid annular plane systolic excursion (TAPSE) and 95% confidence intervals (CIs) measured preoperatively and at 6 months postoperatively. ^*∗*^Between-groups comparison for the 2 surgical techniques. ^†^*p* < 0.0001 versus preoperative.

**Figure 2:**
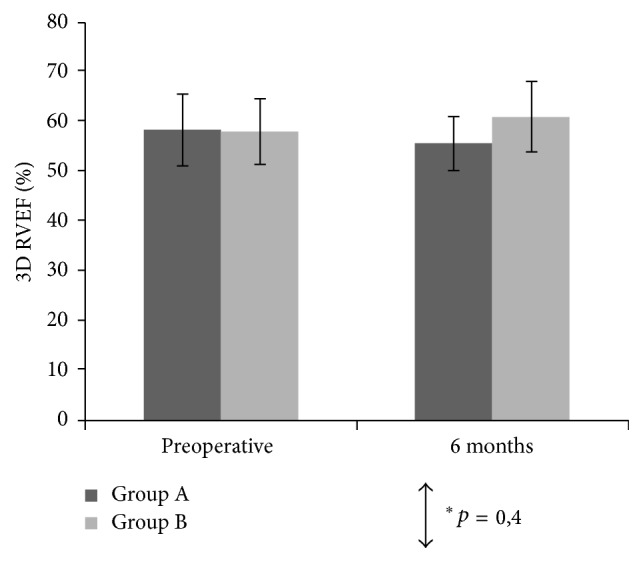
Mean three-dimensional right ventricular ejection fraction (3D RVEF) and 95% confidence intervals (CIs) measured preoperatively and at 6 months postoperatively. ^*∗*^Between-groups comparison for the 2 surgical techniques.

**Figure 3:**
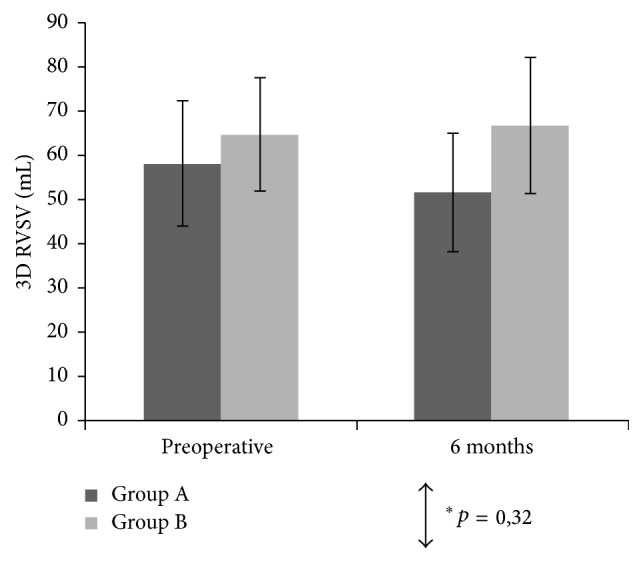
Mean three-dimensional right ventricular stroke volume (3D RVSV) and 95% confidence intervals (CIs) measured preoperatively and at 6 months postoperatively. ^*∗*^Between-groups comparison for the 2 surgical techniques.

All patients in Group A and Group B achieved 6-month residual mitral regurgitation inferior to 1 degree (scale 1 to 4). At least one good quality, three-dimensional right ventricle dataset was acquired in all patients before surgery and 6 months after surgery.  Table 3 and Figures 1, 2, and 3 show the mean values of the two-dimensional and three-dimensional parameters for each step of the study in both groups.

Preoperative right ventricular function was normal in all patients. No significant differences were detected between Group A and Group B about basal right ventricular volumes and function and cross-clamping time. Basal TAPSE was slightly greater in traditional surgery (25.8 mm versus 23.5 mm) but the difference was not significant (*p* = 0.12).

### 3.1. Two-Dimensional Measurements

2D echo showed a postoperative TAPSE decrease in both groups. At follow-up, only Group A showed a significant decrease in TAPSE (preop. 25.8 ± 5.2 mm; postop. 15.2 ± 3.1 mm; *p* < 0.0001).

This difference was statistically significant after adjustment for patients' age, sex, body surface area, and basal TAPSE. Group B showed a less marked TAPSE reduction (preop. 23.5 ± 3.4 mm; postop. 22.2 ± 4.1 mm; *p* = 0.06). Systolic pulmonary arterial pressure showed a similar postoperative fall in both groups.

### 3.2. Three-Dimensional Measurements

3D echocardiography revealed no significant intergroup differences in postoperative changes of end-systolic and end-diastolic right ventricular volumes. In Group B, right ventricular stroke volume and ejection fraction slightly increased after surgery (66.9 ± 15.4 ml postop. versus 64.9 ± 12.8 ml preop. and 60.8 ± 7 ml postop. versus 57.9 ± 6.6 ml preop., resp.). Group A showed a mild reduction of both values (51.9 ± 13.4 ml postop. versus 58.4 ± 14.2 ml preop. and 55.4 ± 5.4 ml postop. versus 58.2 ± 7.2 ml preop., resp.), but such difference did not reach statistical or clinical significance (*p* = 0.4).

## 4. Discussion

Right ventricular function is recognized as an important determinant of several cardiovascular diseases and its importance as a prognostic index after surgery has been widely demonstrated. The accurate postoperative assessment of right ventricular function is crucial and should be based on reliable and reproducible measurements. In particular, right ventricle echocardiographic evaluation has been limited in the past due to the complex structure and anatomy of the right ventricle, but the measurement of tricuspid annulus movement by two-dimensional analysis has been proven to be accurate, feasible, simple, and reproducible in both normal and pathological patients [16]. More accurate right ventricular chamber description independent of any geometrical assumption could be achieved only with the introduction of three-dimensional echocardiography, which allowed the computation in three-dimensional space of the right ventricular volume and function throughout the cardiac cycle [17]. Accordingly, the three-dimensional echo-derived right ventricular ejection fraction could be considered as a parameter of global right ventricular performance, not limited to the individual systolic aspect, such as that described by the tricuspid annulus or by the ventricular free wall movements.

A reduction in TAPSE after cardiac surgery is a well-known phenomenon and has been previously reported in both congenital and acquired diseases [6, 18]. This observation has been interpreted as an isolated worsening of right ventricular performance, without changes in left ventricle parameters or exercise capacity and thus with poor clinical significance. Several hypotheses have been proposed to explain this loss in right ventricular performance detected along the long axis, including cardiopulmonary bypass use [9, 19], geometrical changes of the right ventricular chamber (in association with interventricular septal paradoxical motion [20]), intraoperative ischaemia, right atrial injury due to cannulation procedure [21], poor myocardial protection [22], and extramyocardial causes (pericardial disruption, changes in fossa ovale, and postoperative adherence of the right ventricle to the thoracic wall [10]). In more detail, investigators' attention was particularly addressed to the role played by pericardial injury but definitive conclusions concerning its real contribution failed to be drawn. Unsworth et al. [4] assessed temporal changes in right ventricular two-dimensional features according to the different surgical acts in order to narrow the range of factors that may cause the observed right ventricular functional loss. They demonstrated that peak systolic velocity and TAPSE are reduced within the first 3 minutes after opening the pericardium and hypothesized that the loss of pericardial support, which is fundamental for maintaining right ventricle chamber geometry, makes the right ventricle susceptible to changes when the pericardial constraint itself is lost. These findings support the hypothesis that geometric rather than functional changes happen in the right ventricle during surgery. On the contrary, such theory was not proved by Lindqvist et al. [21], who, by examining the effect of pericardial suture after mitral surgery completion, found that it had no consequences on right ventricular function restoration.

Nevertheless, the assumption of a preeminent right ventricular chamber geometric modification seems to be confirmed by the paper of Tamborini et al. [3] who were able to demonstrate that despite the reduction in right ventricular performance measured after surgery along the ventricular long axis by TAPSE and peak systolic velocity, no associated decrease in three-dimensional right ventricular ejection fraction was found, leading to caution in the interpretation of two-dimensional and Doppler parameters.

Recent findings on the role of pericardial incision in different types of cardiothoracic operations [11] underlined the fact that only in the case of interventions involving complete pericardial opening did the two-dimensional right ventricular systolic function parameters fall one month after surgery, while no changes were detected in robotic assisted, minimal sternotomy or extrapericardial surgical acts. Since it appears quite clear that, among the different hypotheses proposed in order to explain right ventricular postoperative longitudinal decreased contractile performance, pericardial injury and its consequent modification on right ventricle dynamics pattern require greater attention, we tried to investigate whether a different modality of pericardial approach imposed by a minimally invasive intervention could clarify such phenomenon.

In the present study, patients undergoing traditional or minimally invasive surgical mitral valve prolapse repair for chronic severe regurgitation associated with mild pulmonary hypertension or symptoms before the onset of right or left ventricular dysfunction were included [23]. Some possible confounding factors theoretically able to influence postoperative right ventricular systolic fall were eliminated, since the modality of pericardial suture after surgical act completion, cross-clamping, and cardiopulmonary bypass time were comparable in both groups. Only the myocardial protection protocol differed since mixed retro-anterograde blood cold cardioplegia repeated administration was employed in Group A while Group B underwent one-shot antegrade Custodiol solution. Some data available in the medical literature seem to affirm that cardioplegia injection via the coronary sinus fails to fully protect the right ventricle [24], leading consequently to possible subclinical dysfunction. Nevertheless, opinions are far from unanimous as suggested by Kulshrestha et al. [25]. Postoperative right ventricle impairment is subclinical and limited to isolated metabolic changes and this issue appears to be especially valuable in case of normothermic cardioplegia solution [26].

Even considering such possible study limitation, we show (1) that only the long-axis function of the right ventricle is impaired in all patients independently of the surgical approach, while three-dimensional global systolic indexes appear to be substantially unvaried, and (2) that lateral pericardial incision seems to greatly limit right ventricle longitudinal postsurgery function decrease.

Incontestable explications of such findings cannot be proposed, as we can only suggest that a possible aetiology may be retrieved in the anatomical situation of the right ventricle and in its relationship with the posterior surface of visceral pericardium: in fact, by one side, when an anterior pericardial incision is performed, it lies just in front of the anterior wall of the right ventricle and thus we can imagine that, even after pericardial suture, more relevant geometrical changes in right ventricular dynamics occur with respect to what happens in case of a lateral pericardial incision more located in face of the interatrial groove. An alternative explication could lie in the shape itself of the reversed T incision: as it includes a double opening line along the diaphragm (which is directly in relationship with the inferior right ventricular wall), the interaction between this muscle and the right ventricle could be modified, leading to variations in longitudinal right ventricular contractile pattern and consequently to postoperative TAPSE fall.

## Figures and Tables

**Table 1:** Clinical and echocardiographic groups' baseline characteristics.

	Group A (*n* = 17)	Group B (*n* = 17)	*p* value
Age, y	54,82 ± 11,98	50,94 ± 12,20	0.12
Male, % (*n*)	76% (13)	82% (14)	0.25
BSA (m^2^)	1,90 ± 0,22	1,85 ± ,18	0.31
NYHA class			
I	0	0	
II	9	10	0.5
III/IV	8	7	0.43
EuroSCORE 2^*∗*^	0,98%	0,95%	0.42
LVEF	58,6 ± 8.9	59,1 ± 9,1	0.32
TAPSE (mm)	25.8 ± 5,3	23,5 ± 3,4	0.12
PAPs (mmHg)	30,5 ± 2,9	30,9 ± 2,7	0.4
RVEF (3D)	58,2 ± 7.2	57,9 ± 6,6	0.28
RVSV (3D)	58,4 ± 14,2	64,9 ± 12,8	0.09
RVESV (3D)	43,6 ± 17,9	47,7 ± 13,3	0.25
RVEDV (3D)	102 ± 28,6	112,6 ± 21,7	0.08
MVP type			
Posterior leaflet prolapse	17	17	
Anterior leaflet prolapse	2	1	0.14

^*∗*^EuroSCORE 2: the European System for Cardiac Operative Risk Evaluation (2nd version).

BSA: body surface area; NYHA: New York Heart Association; LVEF: left ventricular ejection fraction; TAPSE: tricuspid annular plane systolic excursion; SPAP: systolic pulmonary arterial pressure; RVEF: right ventricular ejection fraction; RVSV: right ventricular stroke volume; RVESV: right ventricular end-systolic volume; RVEDV: right ventricular end-diastolic volume; MVP: mitral valve prolapse.

**Table 2:** Intraoperative groups' characteristics.

	Group A (*n* = 17)	Group B (*n* = 17)	*p* value
CPB time (min)	113 ± 17	131 ± 23	0.07
Cross-clamp time (min)	95 ± 13	112 ± 12	0.08
Complete prosthetic semirigid ring	3	2	0.32
Incomplete band	14	15	0.38
Annular plication	1	0	0.41
Quadrangular resection	6	7	0.42
Triangular resection	9	9	
Sliding plasty	13	17	0.09
Artificial chordae positioning	2	0	0.18
Papillary muscle placation	1	0	0.39

CPB: cardiopulmonary bypass.

**Table 3:** Two-dimensional and three-dimensional echocardiographic parameters measured before and 6 months after surgery.

Variable	Presurgery	Sixth month	*p* within group	*p* between groups
TAPSE (mm)				<0.0001
Group A	25.8 ± 5.3	15.2 ± 3.1	<0.0001	
Group B	23.5 ± 3.4	22.2 ± 4.1	0.06	
*SPAP (mmHg)*				0.5
*Group A*	30.5 ± 2.9	31.4 ± 3,1	0.32	
*Group B*	30.9 ± 2.7	30.7 ± 3.2	0.39	
RVEDV (ml)				0.7
Group A	102 ± 28.6	93 ± 24.1	0.06	
Group B	112.6 ± 21.7	110 ± 21.2	0.8	
RVESV (ml)				0.8
Group A	43.6 ± 17.9	41.7 ± 12.9	0.2	
Group B	47.7 ± 13.3	43 ± 10.7	0.15	
3D RVEF (%)				0.4
Group A	58.2 ± 7.2	55.4 ± 5.4	0.19	
Group B	57.9 ± 6.6	60.8 ± 7	0.21	
3D RVSV (ml)				0.32
Group A	58.4 ± 14.2	51.9 ± 13.4	0.07	
Group B	64.9 ± 12.8	66.9 ± 15.4	0.24	

TAPSE: tricuspid annular plane systolic excursion; SPAP: systolic pulmonary arterial pressure; RVEDV: right ventricular end-diastolic volume; RVESV: right ventricular end-systolic volume; RVEF: right ventricular ejection fraction; RVSV: right ventricular stroke volume.
